# Features and development of *Coot*

**DOI:** 10.1107/S0907444910007493

**Published:** 2010-03-24

**Authors:** P. Emsley, B. Lohkamp, W. G. Scott, K. Cowtan

**Affiliations:** a Department of Biochemistry, University of Oxford, South Parks Road, Oxford OX1 3QU, England; b Department of Medical Biochemistry and Biophysics, Karolinska Institute, SE-171 77 Stockholm, Sweden; c Department of Chemistry, University of California, 1156 High Street, Santa Cruz, CA 95064, USA; d Department of Chemistry, University of York, Heslington, York YO10 5DD, England

**Keywords:** *Coot*, model building

## Abstract

*Coot* is a molecular-graphics application for model building and validation of biological macromolecules. The program displays electron-density maps and atomic models and allows model manipulations such as idealization, real-space refinement, manual rotation/translation, rigid-body fitting, ligand search, solvation, mutations, rotamers and Ramachandran idealization. Furthermore, tools are provided for model validation as well as interfaces to external programs for refinement, validation and graphics. The software is designed to be easy to learn for novice users, which is achieved by ensuring that tools for common tasks are ‘discoverable’ through familiar user-interface elements (menus and toolbars) or by intuitive behaviour (mouse controls). Recent developments have focused on providing tools for expert users, with customisable key bindings, extensions and an extensive scripting interface. The software is under rapid development, but has already achieved very widespread use within the crystallographic community. The current state of the software is presented, with a description of the facilities available and of some of the underlying methods employed.

## Abstract 2

*Coot* is a molecular-graphics program designed to assist in the building of protein and other macromolecular models. The current state of development and available features are presented.

## 1. Introduction

Macromolecular model building using X-ray data is an interactive task involving the iterative application of various optimization algorithms with evaluation of the model and interpretation of the electron density by the scientist. *Coot* is an interactive three-dimensional molecular-modelling program particularly designed for the building and validation of protein structures by facilitating the steps of the process.

In recent years, initial construction of the protein chain has often been carried out using automatic model-building tools such as *ARP*/*wARP* (Langer *et al.*, 2008 ▶), *SOLVE*/*RESOLVE* (Wang *et al.*, 2004 ▶) and more recently *Buccaneer* (Cowtan, 2006 ▶). In consequence, relatively more time and emphasis is placed on model validation than has previously been the case (Dauter, 2006 ▶). The refinement and validation steps become increasingly important and also more time-consuming with lower resolution data. *Coot* aims to provide access to as many of the tools required in the iterative refinement and validation of a macromolecular structure as possible in order to facilitate those aspects of the process which cannot be performed automatically. A primary design goal has been to make the software easy to learn in order to provide a low barrier for scientists who are beginning to work with X-ray data. While this goal has not been met for every feature, it has played a major role in many of the design decisions that have shaped the software.

The principal tasks of the software are the visualization of macromolecular structures and data, the building of models into electron density and the validation of existing models; these will be considered in the next three sections. The remaining sections of the paper will deal with more technical aspects of the software, including interactions with external software, scripting and testing.

## 2. Program design

The program is constructed from a range of existing software libraries and a purpose-written *Coot* library which provides a range of tools specific to model building and visualization. The OpenGL and other graphics libraries, such as the X Window System and GTK+, provide the graphical user-interface functionality, the GNU Scientific Library (GSL) provides mathematical tools such as function minimizers and the Clipper (Cowtan, 2003 ▶) and MMDB (Krissinel *et al.*, 2004 ▶) libraries provide crystallographic tools and data types. On top of these tools are the *Coot* libraries, which are used to manipulate models and maps and to represent them graphically.

**Figure 1:**
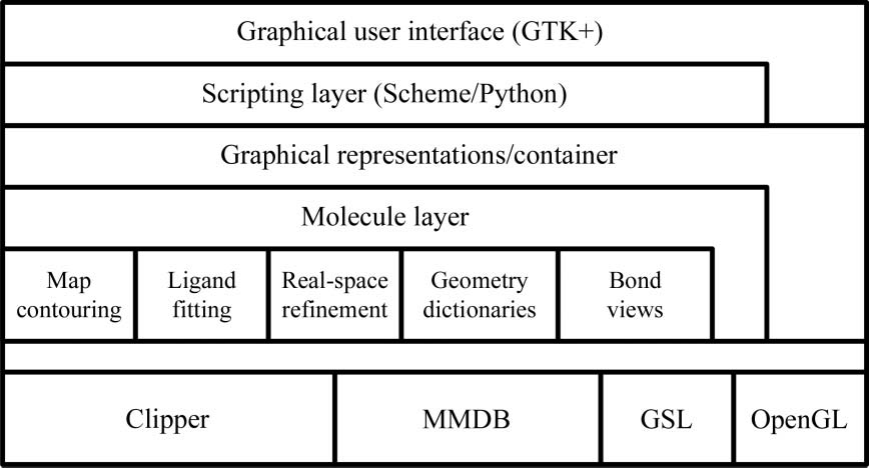
*Coot* architecture, showing the layers of functionality from the user interface down to some of the low-level libraries. The libraries are described in more detail in the main text (§§[Sec sec3.4]3.4, [Sec sec3.5]3.5, [Sec sec4.3]4.3 and [Sec sec4.4]4.4).

Much of this functionality may be accessed from the scripting layer (see §[Sec sec8]8), which allows programmatic access to all of the underlying functionality. Finally, the graphical user interface is built on top of the scripting layer, although in some cases it is more convenient for the graphical user interface to access the underlying classes directly (Fig. 1 ▶).

## 3. Visualization

Coot provides tools for the display of three-dimensional data falling into three classes.

(i) Atomic models (generally displayed as vectors connecting bonded atoms).
(ii) Electron-density maps (generally contoured using a wire-frame lattice).
(iii) Generic graphical objects (including the unit-cell box, noncrystallographic rotation axes and similar).

A user interface and a set of controls allow the user to interact with the graphical display, for example in moving or rotating the viewpoint, selecting the data to be displayed and the mode in which those data are presented.

The primary objective in the user interface as it stands today has been to make the application easy to learn. Current design of user interfaces emphasizes a number of characteristics for a high-quality graphical user interface (GUI). Such characteristics include learnability, productivity, forgiveness (if a user makes a mistake, it should be easy to recover) and aesthetics (the application should look nice and provide a pleasurable experience). When designing the user interface for *Coot*, we aim to respect these issues; however, this may not always be achieved and the GUI often undergoes redesign. Ideally, a user who has a basic familiarity with crystallographic data but who has never used *Coot* before should be able to start the software, display their data and perform some basic manipulations without any instruction. In order for the software to be easy to learn, it is necessary that the core functionality of the software be discoverable, *i.e.* the user should be able to find out how to perform common tasks without consulting the documentation. This may be achieved in any of three ways.

(i) The behaviour is intuitive, *i.e.* the behaviour of user-interface elements can be either anticipated or determined by a few experiments. An example of this is the rotation of the view, which is accomplished by simply dragging with the mouse.
(ii) The behaviour is familiar and consistent, *i.e.* user-interface elements behave in a similar way to other common software. An example of this is the use of a ‘File’ menu containing ‘Open…’ options, leading to a conventional file-selection dialogue.
(iii) The interface is explorable, *i.e.* if a user needs an additional functionality they can find it rapidly by inspecting the interface. An example of this is the use of organized menus which provide access to the bulk of the program functionality. Furthermore, tooltips are provided for most menus and buttons and informative widgets explain their function.

### 3.1. User interface

**Figure 2:**
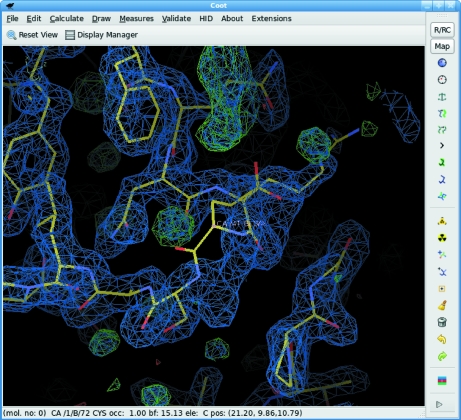
The *Coot* main window. The main display area shows a molecule and electron density. At the top of the window is a menu bar providing access to most of the tools. Commonly used model-manipulation tools are also available through the toolbar on the right. Below the menu bar is an area for user-definable buttons. A status bar is displayed below the three-dimensional canvas.

The main *Coot* user interface window is shown in Fig. 2 ▶ and consists of the following elements.

(i) In the centre of the main window is the three-dimensional canvas, on which the atomic models, maps and other graphical objects are displayed. By default this area has a black background, although this can be changed if desired.
(ii) At the top of the window is a menu bar. This includes the following menus: ‘File’, ‘Edit’, ‘Calculate’, ‘Draw’, ‘Measures’, ‘Validate’, ‘HID’, ‘About’ and ‘Extensions’. The ‘File’, ‘Edit’ and ‘About’ menus fulfill their normal roles. ‘Calculate’ provides access to model-manipulation tools. ‘Draw’ implements display options. ‘Measures’ presents access to geometrical information. ‘Validate’ provides access to validation tools. ‘HID’ allows the human-interface behaviour to be customized. ‘Extensions’ provides access to a range of optional functionalities which may be customized and extended by advanced users. Additional menus can be added by the use of the scripting interface.
(iii) Between the menu bar and the canvas is a toolbar which provides two very frequently used controls: ‘Reset view’ switches between views of the molecules and ‘Display Manager’ opens an additional window which allows individual maps and molecules to be displayed in different ways. This toolbar is customizable, *i.e.* additional buttons can be added.
(iv) On the right-hand side of the window is a toolbar of icons which allow the modification of atomic models. By default these are displayed as icons, although tooltips are provided and text can also be displayed.
(v) Below the canvas is a status bar in which brief text messages are displayed concerning the status of current operations.

The user interface is implemented using the GTK+2 widget stack, although with some work this could be changed in the future.

### 3.2. Controls

User input to the program is primarily *via* mouse and keyboard, although it is also possible to use some dial devices such as the ‘Powermate’ dial. The mouse may be used to select menu options and toolbar buttons in the normal way.

**Figure 3:**
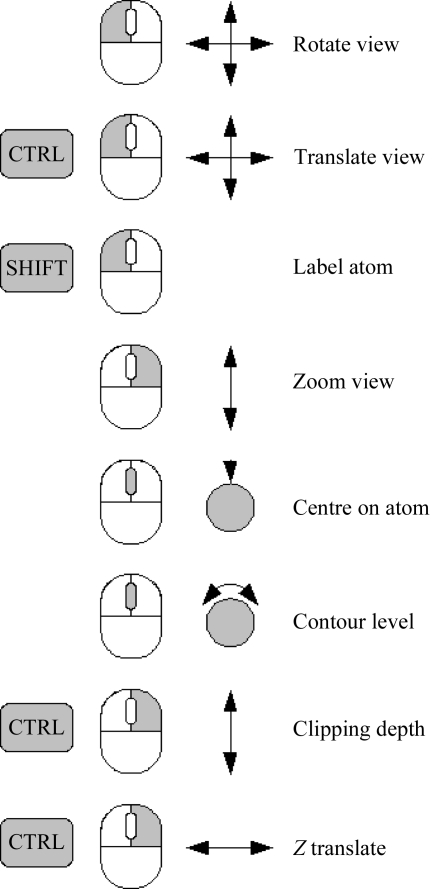
Mouse controls: a schematic mouse is shown with the clicked button in grey. Additional keys to be pressed are shown to the left of the mouse. On the right-hand side is a schematic of the mouse control action together with an explanation.

In addition, the mouse and the keyboard may be used to manipulate the view in the three-dimensional canvas using the controls shown in Fig. 3 ▶.

In a large program there is often tension between software being easy to learn and being easy to use. A program which is easy to use provides extensive shortcuts to allow common tasks to be performed with the minimum user input. Keyboard shortcuts, customizations and macro languages are common examples and are often employed by expert users of all types of software. *Coot* now provides tools for all of these. Much of the functionality of the package is now accessible from both the Python (http://www.python.org) and the Scheme (Kelsey *et al.*, 1998 ▶) scripting languages, which may be used to construct more powerful tools using combinations of existing functions. One example is a function often used after molecular replace­ment which will step through every residue in a protein, replace any missing atoms, find the best-fitting side-chain rotamer and perform real-space refinement. This function is in turn bound to a menu item, although it would also be possible to bind it to a key on the keyboard.

### 3.3. Lighting model

The lighting model used in *Coot* is a departure from the approach adopted in most molecular-graphics software. It is difficult to illustrate a three-dimensional shape in a two-dimensional representation of an object. The traditional approach is to use so-called ‘depth-cueing’: objects closer to the user appear more brightly lit and more distant objects are more like the background colour (usually darker). In the *Coot* model, however, the most brightly lit features are just forward of the centre of rotation. This innovation was accidental, but has been retained because it seemed to provide a more natural image and has generated positive feedback from users once they become accustomed to the new behaviour. It is now possible to offer an explanation for this result.

Depth-cueing is an algorithm which adjusts the colours of graphical objects according to their distance from the viewer. Depth-cueing is used in several ways. When rendering outdoor scenes, it is used to wash out the colours of distant features to simulate the effect of light scattering in the intervening air. When rendering darkened scenes, the same effect can be used to darken distant objects in order to create the effect that the viewer is carrying a light source which illuminates nearer objects more brightly than distant ones. Note that both of these usages assume a ‘first-person’ view: the observer is placed within the three-dimensional environment. This is also borne out in the controls for manipulating the view: when the view is rotated, the whole environment usually rotates about the observer.

However, fitting three-dimensional atomic models to X-­ray data is a different situation. It is not useful to place the observer inside the model and rotate the model around them, not least because the scientist is usually more interested in looking at the molecule or electron density from the outside. As a result, it is normal to rotate the view not about the observer but rather about the centre of the feature being studied. Since the central feature is of most interest, it helps the visualization if it is the brightest entity. To properly light the model in this way is relatively slow, so in *Coot* an approximation is used and the plane perpendicular to the viewer that contains the central feature is most brightly lit.

### 3.4. Atomic model

*Coot* displays the atoms of the atomic models as points on the three-dimensional canvas. If the points are within bonding distance then a line symbolizing a bond is drawn between the atomic points; otherwise the atoms are displayed as crosses. By default the atoms are coloured by element, with carbon yellow, oxygen red, nitrogen blue, sulfur green and hydrogen white. Bonds have two colours, with one half corresponding to each connecting atom. Additional atomic models are distinguished by different colour coding. The colour wheel is rotated and the element colours are adjusted accordingly. However, there is an option to fix the colours for noncarbon elements and the colour-wheel position can be adjusted for each molecule individually. Furthermore, *Coot* allows the user to colour the atomic model by molecule, chain, secondary structure, *B* factor and occupancy. Besides showing atomic models, *Coot* can also display C^α^ (or backbone) chains only. Again the model can be coloured in different modes, by chain, secondary structure or with rainbow colours from the N-terminus to the C-terminus. Currently, *Coot* offers some additional atomic representations in the form of different bond-width or ball-and-stick representation for selected residues.

Information about individual atoms can be visualized in the form of labels. These show the atom name, residue number, residue name and chain identifier. Labels are shown upon Shift + left mouse click or double left mouse click on an atom (the atom closest to the rotation/screen centre can be labelled using the keyboard shortcut ‘l’). This operation not only shows the label beside the atom in the three-dimensional canvas, but also gives more detailed information about the atom, including occupancy, *B* factor and coordinates, in the status bar.

Symmetry-equivalent atoms of the atomic model can be displayed in *Coot* within a certain radius either as whole chains or as atoms within this radius. Different options for colouring and displaying atoms or C^α^ backbone are provided. The symmetry-equivalent models can be labelled as described above. Additionally, the label will provide information about the symmetry operator used to generate the selected model.

Navigation around the atomic models is primarily achieved with a GUI (‘Go To Atom…’). This allows the view to be centred on a particular atom by selection of a model, chain ID, residue number and atom name. Buttons to move to the next or previous residue are provided and are also available *via* keyboard shortcuts (space bar and Shift space bar, respectively). Furthermore, each chain is displayed as an expandable tree of its residues, with atoms that can be selected for centring. Additionally, a mouse can be used for navigation, so a middle mouse click centres on the clicked atom. A keyboard shortcut for the view to be centred on a C^α^ atom of a specific residue is provided by the use of Ctrl-g followed by input of the chain identifier and residue number (terminated by Enter).

All atomic models, in contrast to other display objects, are accessible by clicking a mouse button on an atom centre. This allows, for example, re-centring, selection and labelling of the model.

### 3.5. Electron density

Electron-density maps are displayed using a three-dimensional mesh to visualize the surface of electron-density regions higher than a chosen electron-density value using a ‘marching-cubes’-type algorithm (Lorensen & Cline, 1987 ▶). The spacing of the mesh is dictated by the spacing of the grid on which the electron density is sampled. Since electron-density maps are most often described in terms of structure factors, the sampling can be modified by the user at the point where the electron density is read into the program. The contour level may be varied interactively using the scroll wheel on the mouse (if available) or alternatively by using the keyboard (‘+’ and ‘-’). In most cases this avoids the need for multiple contour levels to be displayed at once, although additional contour levels can be displayed if desired.

The colour of the electron-density map may be selected by the user. By default, the first map read into the program is contoured in blue, with subsequent maps taking successive colour hues around a colour wheel. Difference maps are by default contoured at two levels, one positive and one negative (coloured green and red, respectively).

The electron density is contoured in a box about the current screen centre and is interactively re-contoured whenever the view centre is changed. By default, this box covers a volume extending at least 10 Å in each direction from the current screen centre. This is an appropriate scale for manipulating individual units of a peptide or nucleotide chain and provides good interactive performance, even on older computers. Larger volumes may be contoured on faster machines. A ‘dynamic volume’ option allows the volume contoured to be varied with the current zoom level, so that the contoured region always fills the screen. A ‘dynamic sampling’ option allows the map to be contoured on a subsampled grid (*e.g.* every second or fourth point along each axis). This is useful when using a solvent mask to visualize the packing of the molecules in the crystal.

### 3.6. Display objects

There are a variety of non-interactive display objects which can also be superimposed on the atomic model and electron density. These include the boundaries of the unit cell, an electron-density ridge trace (or skeleton), surfaces, three-dimensional text annotations and dots (used in the *MolProbity* interface). These cannot be selected, but aid in the visualization of features of the electron density and other entities.

### 3.7. File formats

*Coot* recognizes a variety of file formats from which the atomic model and electron density may be read. The differences in the information stored in these various formats mean that some choices have to be made by the user. This is achieved by providing several options for reading electron density and, where necessary, by requesting additional information from the user. The file formats which may be used for atomic models and for electron density will be considered in turn.

In addition to obtaining data from the local storage, it is also possible to obtain atomic models directly from the Protein Data Bank (Bernstein *et al.*, 1977 ▶) by entering the PDB code of a deposited structure. Similarly, in the case of structures for which experimental data have been deposited, the model and phased reflections may both be obtained from the Electron Density Server (Kleywegt *et al.*, 2004 ▶).

#### 3.7.1. Atomic models

Atomic models are read into *Coot* by selecting the ‘Open Coordinates…’ option from the File menu. This provides a standard file selector which may be used to select the desired file. *Coot* recognizes atomic models stored in the following three formats.

(i) Protein Data Bank (PDB) format (with file extension .pdb or .ent; compressed files of this format with extension .gz can also be read). The latest releases provide compatibility with version 3 of the PDB format.
(ii) Macromolecular crystallographic information file (mmCIF; Westbrook *et al.*, 2005 ▶) format (extension .cif).
(iii) *SHELX* result files produced by the *SHELXL* refinement software (extension .res or .ins).

In each case, the unit-cell and space-group information are read from the file (in the case of *SHELXL* output the space group is inferred from the symmetry operators). The atomic model is read, including atom name, alternate conformation code, monomer name, sequence number and insertion code, chain name, coordinates, occupancy and isotropic/anisotropic atomic displacement parameters. PDB and mmCIF files are handled using the MMDB library (Krissinel *et al.*, 2004 ▶), which is also used for internal model manipulations.

#### 3.7.2. Electron density

The electron-density representation is a significant element of the design of the software. *Coot* employs a ‘crystal space’ representation of the electron density, in which the electron density is practically infinite in extent, in accordance with the lattice repeat and cell symmetry of the crystal. Thus, no matter where the viewpoint is located in space density can always be represented. This design decision is achieved by use of the Clipper libraries (Cowtan, 2003 ▶).

The alternative approach is to just display electron density in a bounded box described by the input electron-density map. This approach is simpler and may be more appropriate in some specific cases (*e.g.* when displaying density from cryo-EM experiments or some types of NCS maps). However, it has the limitation that no density is available for symmetry-related molecules and if the initial map has been calculated with the wrong extent then it must be recalculated in order to view the desired regions.

This distinction is important in that it affects how electron-density data should be prepared for use in *Coot*. Files pre­pared for *O* or *PyMOL* may not be suitable for use in *Coot*. In order to read a map file into *Coot*, it should cover an asymmetric unit or unit cell. In contrast, map files prepared for *O* (Jones *et al.*, 1991 ▶) or *PyMOL* (DeLano, 2002 ▶) usually cover a bounded box surrounding the molecule. While it is possible to derive any bounded box from the asymmetric unit, it is not always possible to go the other way; therefore, using map files prepared for other software may lead to unexpected results in some cases, the most common being an incorrect calculation of the standard deviation of the map. If one uses more advanced techniques that involve masking, the electron-density map must have the same symmetry as the associated model molecule.

Electron density may be read into *Coot* either in the form of structure factors (with optional weights) and phases or alternatively in the form of an electron-density map. There are a number of reasons why the preferred approach is to read reflection data rather than a map.

(i) *Coot* can always obtain a complete asymmetric unit of data, avoiding the problems described above.
(ii) Structure-factor files are generally smaller than electron-density maps.
(iii) Some structure-factor files, and in particular MTZ files, provide multiple sets of data in a single file. Thus, it is possible to read a single file and obtain, for example, both best and difference maps.

The overhead in calculating an electron-density map by FFT is insignificant for modern computers.

#### 3.7.3. Reading electron density from a reflection-data file

Two options are provided for reading electron density from a reflection-data file. These are ‘Auto Open MTZ…’ and ‘Open MTZ, mmcif, fcf or phs…’ from the ‘File’ menu.

(i) ‘Auto Open MTZ…’ will open an MTZ file containing coefficients for the best and difference map, automatically select the FWT/PHWT and the DELFWT/DELPHWT pairs of labels and display both electron-density maps. Currently, suitable files are generated by the following software: *Phaser* (Storoni *et al.*, 2004 ▶), *REFMAC* (Murshudov *et al.*, 1997 ▶), *phenix.refine* (Adams *et al.*, 2002 ▶), *DM* (Zhang *et al.*, 1997 ▶), *Parrot* (Cowtan, 2010 ▶), *Pirate* (Cowtan, 2000 ▶) and *BUSTER* (Blanc *et al.*, 2004 ▶).
(ii) ‘Open MTZ, mmcif, fcf or phs…’ will open a reflection-data file in any of the specified formats. Note that *XtalView* .phs files do not contain space-group and cell information: in these cases a PDB file must be read first to obtain the relevant information or the information has to be entered manually. MTZ files may contain many sets of map coefficients and so it is necessary to select which map coefficients to use. In this case the user is provided with an additional window which allows the map coefficients to be selected. The standard data names for some common crystallographic software are provided in Table 1 ▶.

*SHELX* .fcf files are converted to mmCIF format and the space group is then inferred from the symmetry operators.

## 4. Model building

Initial building of protein structures from experimental phasing is usually accomplished by automated methods such as *ARP*/*wARP*, *RESOLVE* (Wang *et al.*, 2004 ▶) and *Buccaneer* (Cowtan, 2006 ▶). However, most of these methods rely on a resolution of better than 2.5 Å and yield more complete models the better the resolution. The main focus in *Coot*, therefore, is the completion of initial models generated by either molecular replacement or automated model building as well as building of lower resolution structures. However, the features described below are provided for cases where an initial model is not available.

### 4.1. Tools for general model building

#### 4.1.1. C^α^ baton mode

Baton building, which was introduced by Kleywegt & Jones (1994 ▶), allows a protein main chain to be built by using a 3.8 Å ‘baton’ to position successive C^α^ atoms at the correct spacing. In *Coot*, this facility is coupled with an electron-density ridge-trace skeleton (Greer, 1974 ▶). Firstly, a skeleton is calculated which follows the ridges of the electron density. The user then selects baton-building mode, which places an initial baton with one end at the current screen centre. Candidate positions for the next α-carbon are highlighted as crosses selected from those points on the skeleton which lie at the correct distance from the start point. The user can cycle through a list of candidate positions using the ‘Try Another’ button or alternatively rotate the baton freely by use of the mouse. Additionally, the length of the baton can be changed to accommodate moderate shifts in the α-­carbon positions. Once a new position is accepted, the baton moves so that its base is on the new α-carbon. In this way, a chain may be traced manually at a rate of between one and ten residues per minute.

#### 4.1.2. C^α^ zone→main chain

Having placed the C^α^ atoms, the rest of the main-chain atoms may be generated automatically. This tool uses a set of 62 high-resolution structures as the basis for a library of main-chain fragments. Hexapeptide and pentapeptide fragments are chosen to match the C^α^ positions of each successive pentapeptide of the C^α^ trace in turn, following the method of Esnouf (1997 ▶), which is similar to that of Jones & Thirup (1986 ▶). The fragments with the best fit to the candidate C^α^ positions are merged to provide a full trace. After this step, one typically performs a real-space refinement of the subsequent main-chain model.

#### 4.1.3. Find secondary structure

Protein secondary-structure elements, including α-helices and β-strands, can be located by their repeating electron-density features, which lead to high and low electron-density values in characteristic positions relative to the consecutive C^α^ atoms. The ‘Find Secondary Structure’ tool performs a six-dimensional rotation and translation search to find the likely positions of helical and strand elements within the electron density. This search has been highly optimized in order to achieve interactive performance for moderately sized structures and as a result is less exhaustive than the corresponding tools employed in automated model-building packages: however, it can provide a very rapid indication of map quality and a starting point for model building.

#### 4.1.4. Place helix here

At low resolution it is sometimes possible to identify secondary-structure features in the electron density when the C^α^ positions are not obvious. In this case, *Coot* can fit an α-helix automatically. This process involves several stages.

(i) A local optimization is performed on the starting position to maximize the integral of the electron density over a 5 Å sphere. This tends to move the starting point close to the helix axis.
(ii) A search is performed to obtain the direction of the helix by integrating the electron density in a cylinder of radius 2.5 Å and length 12 Å. A two-dimensional orientation search is performed to optimize the orientation of the cylinder. This gives the direction of the helix.
(iii) A theoretical α-helical model (including C, C^α^, N and O atoms) is placed in the density in accordance with the position and direction already found. Different rotations of the model around the helix axis must be considered. Each of the resulting models is scored by the sum of the density at the atomic centres. At this stage the direction of the helix is unknown and so both directions are tested.
(iv) Next, a choice is made between the best-fitting models for each helix direction by comparing the electron density at the C^β^ positions. In case neither orientation gives a significant better fit for the C^β^ atoms, both helices are presented to the user.
(v) Finally, attempts are made to extend the helix from the N- and C-termini using ideal ϕ, ψ values.

#### 4.1.5. Place strand here

A similar method is used for placing β-strand fragments in electron density. However, there are three differences compared with helix placement: firstly the initial step is omitted, secondly the length of the fragment (number of residues) needs to be provided by the user and finally the placed fragments are obtained from a database. The first step (optimizing the starting position) is unreliable for strands owing to the smaller radius of the cylinder, *i.e.* main chain, combined with larger density deviations originating from the side chains. Hence, it is omitted and the user must provide a starting position in this case. The integration cylinder used in determining the orientation of the strand has a radius of 1 Å and a length of 20 Å. The ϕ, ψ torsion angles in β-strands in protein deviate from the ideal values, resulting in curved and twisted strands. Such strands cannot be well modelled using ideal values of ϕ and ψ; therefore, candidate strand fragments corresponding to the requested length are taken from a strand ‘database’ (top100 or top500; Word, Lovell, LaBean *et al.*, 1999 ▶) and used in the search.

#### 4.1.6. Ideal DNA/RNA

*Coot* has a function to generate idealized atomic structures of single or double-stranded A-­form or B-form RNA or DNA given a nucleotide sequence. The function is menu-driven and can produce any desired helical nucleic acid coordinates in PDB format with canonical Watson–Crick base pairing from a given input sequence with the click of a single button. Because most DNA and RNA structures are comprised of at least local regions of regular near-ideal helical structural elements, the ability to generate nucleic acid helical models on the fly is of particular value for molecular replacement.

Recently, a collection of short ideal A-form RNA helical fragments generated within *Coot* were used to solve a structurally complex ligase ribozyme by molecular replacement (Robertson & Scott, 2008 ▶). Using *Coot* together with the powerful molecular-replacement program *Phaser* (Storoni *et al.*, 2004 ▶) not only permitted this novel RNA structure to be solved without resort to heavy-atom methods, but several other RNA and RNA/protein complexes were also subsequently determined using this approach (Robertson & Scott, 2007 ▶). Since *Coot* and *Phaser* can be scripted using embedded Python components, an automated and integrated phasing system is amenable for development within the current software framework.

#### 4.1.7. Find ligands

The automatic fitting of ligands into electron-density maps is a frequently used technique that is particularly useful for pharmaceutical crystallographers (see, for example, Williams *et al.*, 2005 ▶). The mechanism in *Coot* addresses a number of ligand-fitting scenarios and is a modified form of a previously described algorithm (Oldfield, 2001 ▶). It is common practice in ‘fragment screening’ to soak different ligands into the same crystal (Blundell *et al.*, 2002 ▶). Using *Coot* one can either specify a region in space or search a whole asymmetric unit for either a single or a number of different ligand types. In the ‘whole-map’ scenario, candidate ligand sites are found by cluster analysis of a residual map. The candidate ligands are fitted in turn to each site (with the candidate orientations being generated by matching the eigenvectors of the ligand to that of the cluster). Each candidate ligand is fitted and scored against the electron density. The best-fitting orientation of the ligand candidates is chosen.

Ligands often contain a number of rotatable bonds. To account for this flexibility, *Coot* samples torsion angles around these rotatable bonds. Here, each rotatable bond is sampled from an independent probability distribution. The number of conformers is under user control and it is recommended that ligands with a higher number of rotatable bonds should be allowed more conformer candidates. Above a certain number of rotatable bonds it is more efficient to use a ‘core + fragment by fragment’ approach (see, for example, Terwilliger *et al.*, 2006 ▶).

### 4.2. Rebuilding and refinement

The rebuilding and refinement tools are the primary means of model manipulation in *Coot* and are all grouped together in the ‘Model/Fit/Refine’ toolset. These tools may be accessed either through a toolbar (which is usually docked on the right-hand side of the main window) or through a separate ‘Model/Fit/Refine’ window containing buttons for each of the toolbar functions.

The core of the rebuilding and refinement tools is the real-space refinement (RSR) engine, which handles the refinement of the atomic model against an electron-density map and the regularization of the atomic model against geometric restraints. Refinement may be invoked both interactively, when executed by the user, and non-interactively as part of some of the automated fitting tools. The refinement and regularization tools are supplemented by a range of additional tools aimed at assisting the fitting of protein chains. These features are discussed below.

### 4.3. Tools for moving existing atoms

#### 4.3.1. Real-space refine zone

The real-space refine tool is the most frequently used tool for the refinement and rebuilding of atomic models and is also incorporated as a final stage in a number of other tools, *e.g.* ‘Add Terminal Residue…’. In interactive mode, the user selects the RSR button and then two atoms bounding a range of monomers (amino acids or otherwise). Alternatively, a single atom can be selected followed by the ‘A’ key to refine a monomer and its neighbours. All atoms in the selected range of monomers will be refined, including any flanking residues. Atoms of the flanking residues are marked as ‘fixed’ but are required to be added to the refinement so that the geometry (*e.g.* peptide bonds, angles and planes) between fixed and moving parts is also optimized.

**Figure 4:**
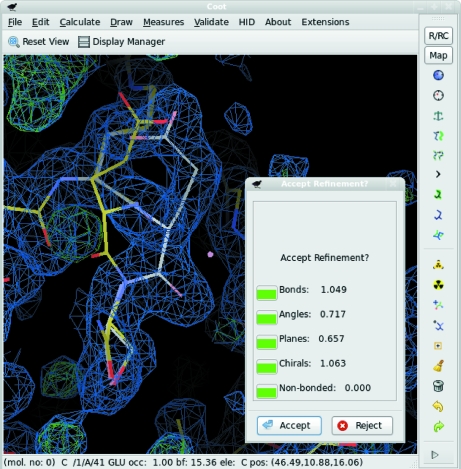
Real-space refinement of a mispositioned residue. The coloured bonds show the original structure. The white bonds show the refined atoms after dragging and refinement. The coloured boxes in the pop-up window indicate how well the new model obeys various geometric restraints. Clicking the ‘Accept’ button will cause the coloured atoms to be moved to the new positions.

The selected atoms are refined against a target consisting of two terms: the first being the atomic number (*Z*) weighted sum of the electron-density values over all the atomic centres and the second being the stereochemical restraints. The progress of the refinement is shown with a new set of atoms displayed in white/pale colours. When convergence is reached the user is shown a dialogue box with a set of χ^2^ scores and coloured ‘traffic lights’ indicating the current geometry scores in each of the geometrical criteria (Fig. 4 ▶). Additionally, a warning is issued if the refined range contains any new *cis*-peptide bonds.

At this stage the user may adjust the model by selecting an atom with the mouse and dragging it, whereby the other atoms will move with the dragged atom. Alternatively, a single atom may be dragged by holding the Ctrl key. As soon as the atoms are released, the selected atoms will refine from the dragged position. Optionally, before the start of refinement atoms may be selected to be fixed during the refinement (in addition to the atoms of the flanking residues).

#### 4.3.2. Sphere refinement

One of the problems with the refinement mode described above is that it only considers a linear range of residues. This can cause difficulties, with some side chains being inappropriately refined into the electron density of neighbouring residues, particularly at lower resolutions. Additionally, a linear residue selection precludes the refinement of entities such as disulfide bonds. Therefore, a new residue-selection mechanism was introduced to address these issues: the so-called ‘Sphere Refinement’. This mode selects residues that have atoms within a given radius of a specified position (typically within 4 Å of the centre of the screen). The selected residues are matched to the dictionary and any user-defined links (typically from the mon_lib_list.cif in the *REFMAC* dictionary), *e.g.* disulfide bonds, glycosidic linkages and formylated lysines. If such links are found and the (supposedly) bonded atoms are within 3 Å of each other then these extra link restraints are added into the refinement.

#### 4.3.3. Ramachandran restraints

At lower resolution it is sometimes difficult to obtain an acceptable fit of the model to the density and at the same time achieve a Ramachandran plot of high quality (most residues in favourable regions and less than 1% outliers). If a Ramachandran score is added to the target function then the Ramachandran plot can be improved.

The analytical form for torsion gradients (∂θ/∂*x*_1_ and so on) for each of the *x*, *y*, *z* positions of the four atoms contributing to the torsion angle has been reported previously (Emsley & Cowtan, 2004 ▶) (in the case of Ramachandran restraints, the θ torsions will be ϕ and ψ). The extension of the torsion gradients for use as Ramachandran restraints is performed in the following manner.

Firstly, two-dimensional log Ramachandran plots *R* are generated as tables (one for each of the residue types Pro, Gly and non-Pro or Gly). Where the Ramachandran probability becomes zero the log probability becomes infinite and so it is replaced by values which become increasingly negative with distance from the nearest nonzero value. This provides a weak gradient in the disallowed regions towards the nearest allowed region. The log Ramachandran plot provides the following values and derivatives:
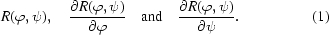
The derivative of *R* with respect to the coordinates is required for the addition into the target geometry and is generated as
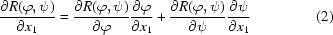
(and so on for each of the *x*, *y*, *z* positions of the atoms in the torsion).

Adding a Ramachandran score to the geometry target function is not without consequences. The Ramachandran plot has for a long time been used as a validation criterion, therefore if it is used in geometry optimization it becomes less informative as a validation metric. Kleywegt & Jones (1996 ▶) included the Ramachandran plot in the restraints during refinement using *X-PLOR* (Brünger, 1992 ▶) and reported that the number of Ramachandran outliers was reduced by about a third using moderate force constants. However, increasing the force constants by over two orders of magnitude only marginally decreased the number of outliers. As a result, Kleywegt and Jones note that the Ramachandran plot retains significant value as a validation tool even when it is also used as a restraint. Using the Ramachandran restraints as implemented in *Coot* with the default weights, the number of out­liers can be reduced from around 10% to 5% (typical values).

#### 4.3.4. Regularize zone

The ‘Regularize Zone’ option functions in the same way as ‘Real-Space Refine Zone’ except that in this case the model is refined with respect to stereochemical restraints but without reference to any electron density.

#### 4.3.5. Rigid-body fit zone

The ‘Rigid-Body Fit Zone’ option also follows a similar interface convention to the other refinement options. A range of atoms are selected and the orientation of the selected group is refined to best fit the density. In this case the density is the only contributor to the target function, since the geometry of the fragment is not altered. No constraints are placed on the bonding atoms. If atoms are dragged after refinement, no further refinement is performed on the fragment.

#### 4.3.6. Rotate/translate zone

Using this tool, the selected residue selection can be translated and rotated either by dragging it around the screen or through the use of user-interface sliders. No reference to the map is made. The rotation centre can be specified to be either the last atom selected or the centre of mass of the fragment rotated. Additionally, a selection of the whole chain or molecule can be transformed.

#### 4.3.7. Rotamer tools

Four tools are available for the fitting of amino-acid side chains. For a side chain whose amino-acid type is already correctly assigned, the best rotamer may be chosen to fit the density either automatically or manually. If the automatic option is chosen then the side-chain rotamer from the *MolProbity* library (Lovell *et al.*, 2000 ▶) which gives rise to the highest electron-density values at the atomic centres is selected and rigid-body refined (this includes the main-chain atoms of the residues). Otherwise, the user is presented with a list of rotamers for that side-chain type sorted by frequency in the database. The user can then scroll through the list of rotamers using either the keyboard or user-interface buttons to select the desired rotamer. Rotamers are named according to the *MolProbity* system. Briefly, the χ angles are given letters according to the torsion angle: ‘t’ for approximately 180°, ‘p’ for approximately 60° and ‘m’ for approximately −60° (Lovell *et al.*, 2000 ▶).

The other two options (‘Mutate & Auto Fit’ and ‘Simple Mutate’) allow the amino-acid type to be assigned or changed. The ‘Mutate & Auto Fit Rotamer’ option allows an amino-acid type to be selected from a list and then immediately performs the autofit rotamer operation as above. The ‘Simple Mutate’ option changes the amino-acid type and builds the side-chain atoms in the most frequently occurring rotamer without further refinement.

#### 4.3.8. Torsion editing (‘Edit Chi Angles’, ‘Edit Backbone Torsions’, ‘Torsion General’)

*Coot* has different tools for editing the main-chain and side-chain (or ligand) torsion angles. The main-chain torsion angles, namely ϕ and ψ, can be edited using ‘Edit Backbone Torsion…’. With two sliders, the peptide and carbonyl torsion angles can be adjusted. A separate window showing the Ramachandran plot with the two residues forming the altered peptide bond is displayed with the position of the residues updated as the angles change. Side-chain (or ligand) torsion angles must be defined prior to editing. Either the user manually defines the four atoms forming the torsion angle (‘Torsion General’) or the torsion angles are determined automatically and the user selects the one to edit. In the latter case the bond around which the selected torsion angle is edited is visually marked. Using the mouse, the angle can then be rotated freely.

#### 4.3.9. Other protein tools (‘Flip peptide’, ‘Side Chain 180° Flip’, ‘Cis→Trans’)

There are three other tools to perform common corrections to protein models. ‘Flip peptide’ rotates the planar atoms in a peptide group through 180° about the vector joining the bounding C^α^ atoms (Jones *et al.*, 1991 ▶). ‘Side Chain 180° Flip’ rotates the last torsion of a side chain through 180° (*e.g.* to swap the OD1 and ND2 side-chain atoms of Asn). ‘Cis→Trans’ shifts the torsion of the peptide bond through 180°, thereby changing the peptide bond from *trans* to *cis* and *vice versa*.

### 4.4. Tools for adding atoms to the model

#### 4.4.1. Find waters

The water-finding mechanism in *Coot* uses the same cluster analysis as is used in ligand fitting. However, only those clusters below a certain volume (by default 4.2 Å^3^) are considered as candidate sites for water molecules. The centre of each cluster is computed and a distance check is then made to the potential hydrogen-bond donors or receptors in the protein molecule (or other waters). The distance criteria for acceptable hydrogen-bond length are under user control. Additionally, a test for acceptable sphericity of the electron density is performed.

#### 4.4.2. Add terminal residue

The *MolProbity* ϕ, ψ distribution is used to generate a set of randomly selected ϕ, ψ pairs.

To build additional residues at the N- and C-termini of protein chains, the *MolProbity* ϕ, ψ distribution is used to generate a set of positions of the N, C^α^, O and C atoms of the next two residues. The conformation of these new atoms is then scored against the electron-density map and recorded. This procedure is carried out a number of times (by default 100). The best-fitting conformation is offered as a candidate to the user (only the nearest of the two residues is kept).

#### 4.4.3. Add alternate conformation

Alternate conformations are generated by splitting the residue into two sets of conformations (*A* and *B*). By default all atoms of the residue are split, or alternatively only the C^α^ and side-chain atoms are divided. If the residue chosen is a standard protein residue then the rotamer-selection dialogue described above is also shown, along with a slider to specify the occupancy of the new conformation.

#### 4.4.4. Place atom at pointer

This is a simple interface to place a typed atom at the position of the centre of the screen. It can place additional water or solvent molecules in un­modelled electron-density peaks and is used in conjunction with the ‘Find blobs’ tool, which allows the largest unmodelled peaks to be visited in turn.

### 4.5. Tools for handling noncrystallographic symmetry (NCS)

Noncrystallographic symmetry (NCS) can be exploited during the building of an atomic model and also in the analysis of an existing model. *Coot* provides five tools to help with the building and visualization of NCS-related molecules.

**Figure 5:**
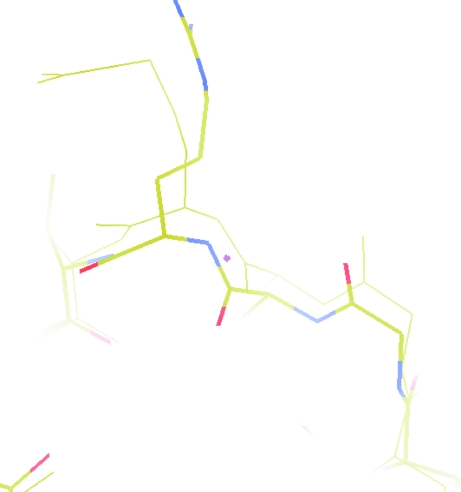
A model with an NCS ghost. The thick bonds represent the atoms in one chain of the protein. The thin bonds represent an NCS-related chain transformed to superpose on the first chain. At the bottom of the screen the atoms coincide; at the top the main chain deviates and a side chain is in a different conformation.

**Figure 6:**
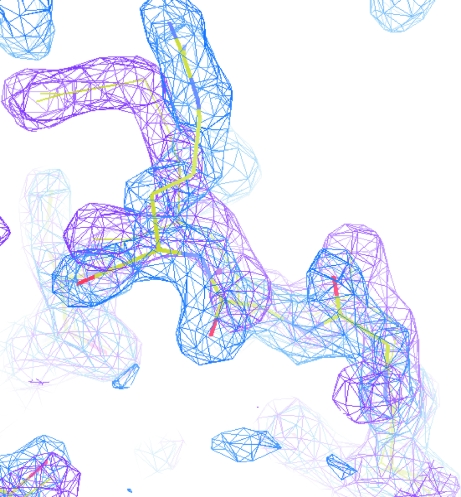
Electron density with NCS map for the same model and in the same orientation as the previous figure. The blue density is for the original chain. The magenta contour represents the electron density for the NCS-related chain transformed back onto the original chain and clearly showing the differences.

(i) NCS ghost molecules. In order to visualize the simi­larities and differences between NCS-related molecules, a ‘ghost’ copy of any or all NCS-related chains may be superimposed over a specific chain in the model. The ‘ghost’ copies are displayed in thin lines and coloured differently, as well as uniformly, in order to distinguish them from the original. The superposition may be performed automatically by secondary-structure matching (Krissinel & Henrick, 2004 ▶) or by least-squares superposition. An example of an NCS ghost molecule is shown in Fig. 5 ▶.
(ii) NCS maps. The electron density of NCS-related molecules can be superimposed in order to allow differences in the electron density to be visualized. This is achieved by transforming the coordinates of the three-dimensional contour mesh, rather then the electron density itself, in order to provide good interactive performance. The operators are usually determined with reference to an existing atomic model which obeys the same NCS relationships. An example of an NCS map is shown in Fig. 6 ▶.
(iii) NCS-averaged maps. In addition to viewing NCS-related copies of the electron density, the average density of the related regions may be computed and viewed. In noisy maps this can provide a clearer starting point for model building.
(iv) NCS rebuilding. When building an atomic model of a molecule with NCS, it is often more convenient to work on one chain and then replicate the changes made in every NCS-related copy of that chain (at least in the early stages of model building). This can be achieved by selecting two related chains and replacing the second chain in its entirety, or in a specific residue range, with an NCS-transformed copy of the first chain.
(v) NCS ‘jumping’. The view centre jumps to the next NCS-related peer chain and at the same time the NCS operators are taken into account so that the relative view remains the same. This provides a means for rapid visual comparison of NCS-related entities.

## 5. Validation

*Coot* incorporates a range of validation tools from the com­parison of a model against electron density to comprehensive geometrical checks for protein structures and additional tools specific to nucleotides.

It also provides convenient interfaces to external validation tools: most notably the *MolProbity* suite (Davis *et al.*, 2007 ▶), but also to the *REFMAC* refinement software (Murshudov *et al.*, 1997 ▶) and dictionary (Vagin *et al.*, 2004 ▶).

**Figure 7:**
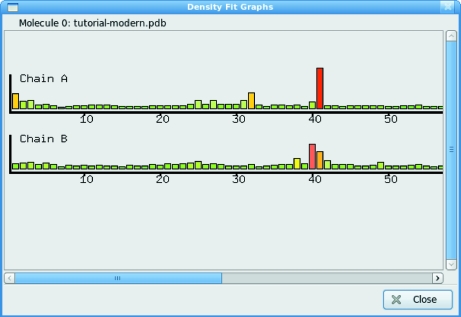
A typical validation graph. Bars represent individual residues in a chain, with an indication of quality for the residue being given by both the size and colour of the bar. The plot is interactive, *i.e.* clicking on a bar takes the user to the corresponding residue.

Many of the internal validation tools provide a uniform interface in the form of colour-coded bar charts, for example the ‘Density Fit Analysis’ chart (Fig. 7 ▶). This window contains one bar chart for each chain in the structure. Each chart contains one bar for each residue in the chain. The height and colour of the bar indicate the model quality of the residue, with small green bars indicating a good or expected/conventional conformation and large red bars indicating poor-quality or ‘unconventional’ residues. The chart is active, *i.e.* on moving the pointer over the bar tooltips provide relevant statistics and clicking on a bar changes the view in the main graphics window to centre on the selected residue. In this way, a rapid overview of model quality is obtained and problem areas can be investigated. In order to obtain a good structure for sub­mission, the user may simply cycle though the validation options, correcting any problems found.

The available validation tools are described in more detail in the following sections.

### 5.1. Ramachandran plot

**Figure 8:**
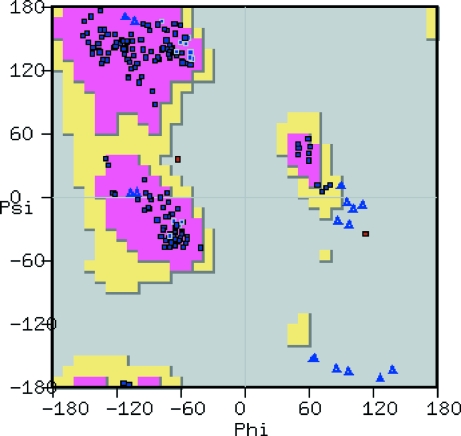
Screenshot of a classical Ramachandran plot showing all residues, with the axes defining the ϕ and ψ angles (angles in degrees). Preferred regions are coloured in pink, allowed regions in yellow and the background in grey for disallowed regions. Standard residues are shown as dark blue squares, Pro residues as light blue squares and Gly residues as light blue open triangles. Residues in the disallowed regions are coloured red.

The Ramachandran plot tool (Fig. 8 ▶) launches a new window in which the Ramachandran plot for the active molecule is displayed. A data point appears in this plot for each residue in the protein, with different symbols distinguishing Gly and Pro residues. The background of the plot shows frequency data for Ramachandran angles using the Richardsons’ data (Lovell *et al.*, 2003 ▶).

The plot is interactive: clicking on a data point moves the view in the three-dimensional canvas to centre on the corresponding residue. Similarly, selecting an atom in the model highlights the corresponding data point. Moving the mouse over a data point corresponding to a Gly or Pro residue causes the Ramachandran frequency data for that residue type to be displayed.

### 5.2. Kleywegt plot

**Figure 9:**
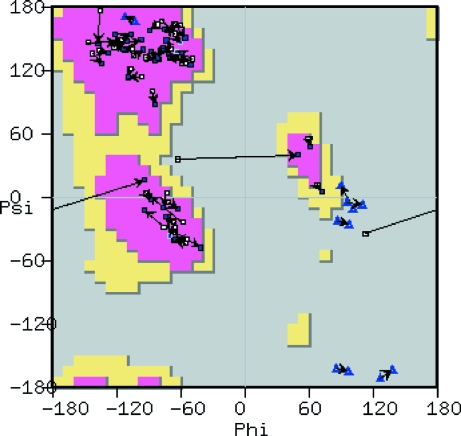
Screenshot of a Kleywegt plot (Kleywegt, 1996 ▶) showing Ramachandran differences between two NCS-related chains by connecting lines (angles in degrees). Labels, colouring and symbols are as in the previous figure. Arrows link NCS-related residues.

The Kleywegt plot (Kleywegt, 1996 ▶; Fig. 9 ▶) is a variation of the Ramachandran plot that is used to highlight NCS differences between two chains. The Ramachandran plot for two chains of the protein is displayed, with the data points of NCS-related residues in the two chains linked by a line for the top 50 (default) most different ϕ, ψ angles. Long lines in the corresponding figure correspond to significant differences in backbone conformation between the NCS-related chains.

### 5.3. Incorrect chiral volumes

Dictionary definitions of monomers can contain descriptions of chiral centres. The chiral centres are described as ‘positive’, ‘negative’ or ‘both’. *Coot* can compare the residues in the protein structure to the dictionary and identify outliers.

### 5.4. Unmodelled blobs

The ‘Unmodelled Blobs’ tool finds candidate ligand-binding sites (as described above) without trying to fit a specific ligand.

### 5.5. Difference-map peaks

Difference maps can be searched for positive and negative peaks. The peak list is then sorted on peak height and filtered by proximity to higher peaks (*i.e.* only peaks that are not close to previous peaks are identified).

### 5.6. Check/delete waters

Waters can be validated using several criteria, including distance from hydrogen-bond donors or acceptors, temperature factor or electron-density level. Waters that do not pass these criteria are identified and presented as a list or automatically deleted.

### 5.7. Check waters by difference map variance

This tool is used to identify waters that have been placed in density that should be assigned to other atoms or molecules. The difference map at each water position is analysed by generating 20 points on each sphere at radii of 0.5, 1.0 and 1.5 Å and the electron-density level at each of these points is found by cubic interpolation. The mean and variance of the density levels is calculated for each set of points. If, for example, a water was misplaced into the density for a glycerol then (given an isotropic density model for the water molecule) the difference map will be anisotropic because there will be unaccounted-for positive density along the bonds to the other atoms in the glycerol. There may also be some negative density in a perpendicular direction as the refinement program tries to compensate for the additional electron density. The variances are summed and compared with a reference value (by default 0.12 e^2^ Å^−6^). Note that it only makes sense to run this test on a difference map generated by reciprocal-space refinement (for example, from *REFMAC* or *phenix.refine*) that included temperature-factor refinement.

### 5.8. Geometry analysis

The geometry (bonds, angles, planes) for each residue in the selected molecule is compared with dictionary values (typically provided by the mmCIF *REFMAC* dictionary). Torsion-angle deviations are not analysed (as there are other validation tools for these; see §[Sec sec5.9]5.9).

The statistic displayed in the geometry graph is the average *Z* value for each of the geometry terms for that residue (peptide-geometry distortion is shared between neighbouring residues). The tooltip on the geometry graph describes the geometry features giving rise to the highest *Z* value.

### 5.9. Peptide ω analysis

This is a validation tool for the analysis of peptide ω torsion angles. It produces a graph marking the deviation from 180° of the peptide ω angle. The deviation is assigned to the residue that contains the C and O atoms of the peptide link, thus peptide ω angles of 90° are very poor. Optionally, ω angles of 0° can be considered ideal (for the case of intentional *ci*s-peptide bonds).

### 5.10. Temperature-factor variance analysis

The variance of the temperature factors for the atoms of each residue is plotted. This is occasionally useful to highlight misbuilt regions. In a badly fitting residue, reciprocal-space refinement will tend to expand the temperatures factors of atoms in low or negative density, resulting in a high variance. However, residues with long side chains (*e.g.* Arg or Lys) often naturally have substantial variance, even though the atoms are correctly placed, which causes ‘noise’ in this graph. This shortcoming will be addressed in future developments. H atoms are ignored in temperature-factor variance analysis.

### 5.11. Gln and Asn *B*-factor outliers

This is another tool that analyses the results of reciprocal-space refinement. A measure *z* is computed that is half of the difference of the temperature factor between the NE2 and OE1 atoms (in the case of Gln) divided by the standard deviation of the temperature factors of the remaining atoms in the residue. Our analysis of high-resolution structures has shown that when *z* is greater than +2.25 there is a more than 90% chance that OE1 and NE2 need to be flipped (P. Emsley, unpublished results).

### 5.12. Rotamer analysis

The rotamer statistics are generated from an analysis of the nearest conformation in the *MolProbity* rotamer probability distribution (Lovell *et al.*, 2000 ▶) and displayed as a bar chart. The height of the bar in the graph is inversely proportional to the rotamer probability.

### 5.13. Density-fit analysis

The bars in the density-fit graphs are inversely proportional to the average *Z*-weighted electron density at the atom centres and to the grid sampling of the map (*i.e.* maps with coarser grid sampling will have lower bars than a more finely gridded map, all other things being equal). Accounting for the grid sampling allows lower resolution maps to have an informative density-fit graph without many or most residues being marked as worrisome owing to their atoms being in generally low levels of density.

### 5.14. Probe clashes

‘Probe Clashes’ is a graphical representation of the output of the *MolProbity* tools *Reduce* (Word, Lovell, Richardson *et al.*, 1999 ▶), which adds H atoms to a model (and thereby provides a means of analyzing potential side-chain flips), and *Probe* (Word, Lovell, LaBean *et al.*, 1999 ▶), which analyses atomic packing. ‘Contact dots’ are generated by *Probe* and these are displayed in *Coot* and coloured by the type of interaction.

### 5.15. NCS differences

The graph of noncrystallographic symmetry differences shows the r.m.s. deviation of atoms in residues after the transformation of the selected chain to the reference chain has been applied. This is useful to highlight residues that have unusually large differences in atom positions (the largest differences are typically found in the side-chain atoms).

## 6. Model analysis

### 6.1. Geometric measurements

Geometric measurements can be performed on the model and displayed in a three-dimensional view using options from the ‘Measures’ menu. These measurements include bond lengths, bond angles and torsion angles, which may be selected by clicking successively on the atoms concerned. It is also possible to measure the distance of an atom to a least-squares plane defined by a set of three or more other atoms. The ‘Environment Distances’ option allows all neighbours within a certain distance of any atom of a chosen residue to be displayed. Distances between polar neighbours are coloured differently to all others. This is particularly useful in the initial analysis of hydrogen bonding.

### 6.2. Superpositions

It is often useful to compare several related molecules which are similar in terms of sequence or fold. In order to do this the molecules must be placed in the same position and orientation in space so that the differences may be clearly seen. Two tools are provided for this purpose.

(i) *SSM* superposition (Krissinel & Henrick, 2004 ▶). *Secondary Structure Matching* (*SSM*) is a tool for superposing proteins whose fold is related by fitting the secondary-structure elements of one protein to those of the other. This approach is automatic and does not rely on any sequence identity between the two proteins. The superposition may include a complete structure or just a single chain.
(ii) LSQ superposition. Least-squares (LSQ) superposition involves finding the rotation and translation which minimizes the distances between corresponding atoms in the two models and therefore depends on having a predefined correspondence between the atoms of the two structures. This approach is very fast but requires that a residue range from one structure be specified and matched to a corresponding residue range in the other structure.

## 7. Interaction with other programs

In addition to the built-in tools, *e.g.* for refinement and validation, *Coot* provides interfaces to external programs. For refinement, interfaces to *REFMAC* and *SHELXL* are pro­vided. Validation can be accomplished by interaction with the programs *Probe* and *Reduce* from the *MolProbity* suite. Furthermore, interfaces for the production of publication-quality figures are provided by communication with the (molecular) graphics programs *CCP*4*mg*, *POV-Ray* and *Raster*3*D*.

### 7.1. *REFMAC*

*Coot* provides a dialogue similar to that used in *CCP*4*i* for running *REFMAC* (Murshudov *et al.*, 2004 ▶). *REFMAC* is a program from the *CCP*4 suite for maximum-likelihood-based macromolecular refinement. Once a round of interactive model building has finished, the user can choose to use *REFMAC* to refine the current model. Reflections for the refinement are either used from the MTZ file from which the currently displayed map was calculated or can be acquired from a selected MTZ file. Most *REFMAC* parameters are set as defaults; however, some can be specified in the GUI, such as the number of refinement cycles, twin refinement and the use of NCS. Once *REFMAC* has terminated, the newly generated (refined) model and MTZ file from which maps are generated are automatically read in (and displayed). If *REFMAC* detected geometrical outliers at the end of the refinement, an interactive dialogue will be presented with two buttons for each residue containing an outlier: one to centre the view on the residue and the other to carry out real-space refinement.

### 7.2. *SHELXL*

For high-resolution refinement, *SHELXL* can be used directly from *Coot*. A new SHELXL.ins file can be generated from a SHELXL.res file including any manipulations or additions to the model. Additional parameters may be added to the file or it can be edited in a GUI. Once refinement in *SHELXL* is finished, the refined coordinate file is read in and displayed. The resulting reflections file (.fcf) is converted into an mmCIF file, after which it is read in and the electron density is displayed. An interactive dialogue of geometric out­liers (disagreeable restraints and other problems discovered by *SHELXL*) can be displayed by parsing the .lst output file from *SHELXL*.

### 7.3. *MolProbity*

*Coot* interacts with programs and data from the *Mol­Probity* suite in a number of ways, some of which have already been described. In addition, *MolProbity* can provide *Coot* with a list of possible structural problems that need to be addressed in the form of a ‘to-do chart’ in either Python or Scheme format; this can be read into *Coot* (‘Calculate’→‘Scripting…’).

### 7.4. *CCP*4*mg*

Coot can write *CCP*4*mg* picture-definition files (Potterton *et al.*, 2004 ▶). These files are human-readable and editable and define the scene displayed by *CCP*4*mg*. Currently, the view and all displayed coordinate models and maps are described in the *Coot*-generated definition file. Hence, the displayed scene in *Coot* when saving the file is identical to that in *CCP*4*mg* after reading the picture-definition file. For convenience, a button is provided which will automatically produce the picture-definition file and open it in *CCP*4*mg*.

### 7.5. *Raster*3*D*/*POV-Ray*

*Raster*3*D* (Merritt & Bacon, 1997 ▶) and *POV-Ray* (Persistence of Vision Pty Ltd, 2004 ▶) are commonly used programs for the production of publication-quality figures in macromolecular crystallography. *Coot* writes input files for both of these programs to display the current view. These can then be rendered and ray-traced by the external programs either externally or directly within *Coot* using ‘default’ parameters. The resulting images display molecular models in ball-and-stick representation and electron densities as wire frames.

## 8. Scripting

Most internal functions in *Coot* are accessible *via* a SWIG (Simplified Wrapper and Interface Generator) interface to the scripting languages Python (http://www.python.org) and Guile (a Scheme interpreter; Kelsey *et al.*, 1998 ▶; http://www.gnu.org/software/guile/guile.html). *Via* the same interface, some of *Coot*’s graphics widgets are available to the scripting layer (*e.g.* the main menu bar and the main toolbar). The availability of two scripting interfaces allows greater flexibility for the user as well as facilitating the interaction of *Coot* with other applications.

In addition to the availability of *Coot*’s internal functions, the scripting interface is enriched by a number of provided scripts (usually available in both scripting languages). Some of these scripts use GUIs, either through use of the *Coot* graphics widgets or *via* the GTK+2 extensions of the scripting lan­guages. A number of available scripts and functions are made available in an extra ‘Extensions’ menu. Scripting not only provides the user with the possibility of running internal *Coot* functions and scripts but also that of reading and writing their own scripts and customizing the menus.

## 9. Building and testing

When *Coot* was made available to the public, three initial considerations were that it should be cross-platform, robust and easy to install. These considerations continue to be a challenge. To assist in meeting them, an automated scheduled build-and-test system has been developed, thus enabling almost constant deployment of the pre-release software.

The subversion version-control system (http://svnbook.red-bean.com/) is used to manage source-code revisions. An ‘integration machine’ checks out the latest source code several times per hour, compiles the software and makes a source-code tar file. Less frequently, a heterogeneous array of build machines copies the source tar file and compiles it for the host architecture. After a successful build, the software is run against a test suite and only if the tests are passed is the software bundled and made available for download from the web site. All the build and test logs are made available on the *Coot* web site. Fortunately, users of the pre-release code seem to report problems without undue exasperation. It is the aim of the developers to respond rapidly to such reports.

### 9.1. Computer operating-system compatibility

*Coot* is released under the GNU General Public License (GPL) and depends upon many other GPL and open-source software components. *Coot*’s GUI and graphical display are based on rather standard infrastructure, including the X11 windowing system, OpenGL and associated software such as the cross-platform GTK+2 stack derived from the GIMP project. In addition, *Coot* depends upon open-source crystallographic software components including the Clipper libraries (Cowtan, 2003 ▶), the MMDB library (Krissinel *et al.*, 2004 ▶), the *SSM* library (Krissinel & Henrick, 2004 ▶) and the *CCP*4 libraries. In principle, *Coot* and its dependencies can be in­stalled on any modern GNU/Linux or Unix platform without fanfare. A Windows-based version of *Coot* is also available.

### 9.2. *Coot* on GNU/Linux

Compiling and installing *Coot* on the GNU/Linux operating system is probably the most straightforward option. GNU/Linux is in essence a free software/open-source collaborative implementation of the Unix operating system that is compatible with most computer hardware. *Coot*’s infrastructural dependencies, such as GTK+2 and other GNU libraries, as well as all of its crystallographic software dependencies, were selected with portability in mind. Most of the required dependencies are either installed with the GNOME desktop or are readily available for installation *via* the package-management systems specific to each distribution.

It is possible that in future *Coot* (along with all its dependencies) will be made available *via* the official package-distribution systems for several of the major GNU/Linux distributions. When an end-user chooses to install the *Coot* package, all of *Coot*’s required dependencies will be installed along with it in a simple and painless procedure.

An official *Coot* package currently exists in the Gentoo distribution (maintained by Donnie Berkholz), a Fedora package (maintained by Tim Fenn) is under development at the time of writing and unofficial Debian and rpm *Coot* packages are also available. Binary *Coot* releases for the most popular GNU/Linux platforms are available from the *Coot* website: http://www.ysbl.york.ac.uk/~emsley/software/binaries/.

Additional information on installing *Coot* on GNU/Linux, either as a pre-compiled binary or from source code, is available on the *Coot* wiki: http://strucbio.biologie.uni-konstanz.de/ccp4wiki/index.php/COOT.

### 9.3. Coot on Apple’s Mac OS X

With the release of Apple’s Mac OS X, a Unix-based operating system, it became possible to use most if not all of the standard crystallographic software on Apple computers. OS X does not natively use the X11 windowing system, but rather a proprietary windowing technology called Quartz. This system has some benefits over X11, but does not support X11-based Unix software. However, the X11 windowing system can be run within OS X (in rootless mode) and as of OS X version 10.5 this has become a default option and operates in a reasonably seamless manner.

Unlike GNU/Linux, Apple does not provide the X11-based dependencies (GTK+2, GNOME libraries) and many of the other open-source components required to install and run *Coot*. However, third-party package-management systems have appeared to fill this gap, having made it their mission to port essentially all of the most important software that is freely available to users of other Unix-based systems to OS X. The two most popular package-management systems are *Fink* and *MacPorts*. Of these, *Fink* makes available a larger collection of software that is of use to scientists, including a substantial collection of crystallographic software. For that reason, *Fink* has been adopted as the preferred option for installing *Coot* on Mac OS X. *Fink* uses many of the same software tools as the Debian GNU/Linux package-management system and provides a convenient front-end.

In practice, this requires the end user to do three things in preparation for installing *Coot* under OS X.

(i) Install Apple’s X-code Developer tools. This is a free gigabyte-sized download available from Apple.
(ii) Install the very latest version of X11. This is crucial, as many bug fixes are required to run *Coot*.
(iii) Install the third-party package-management system *Fink* and enable the ‘unstable’ software tree to obtain access to the latest software.

*Coot* may then be installed through *Fink* with the command fink install coot.

### 9.4. *Coot* on Microsoft Windows

Since Microsoft Windows operating systems are the most widely used computer platform, a *Coot* version which runs on Microsoft Windows has been made available (*WinCoot*). All of *Coot*’s dependencies compile readily on Windows systems (although some require small adjustments) or are available as GPL/open-source binary downloads. The availability of GTK+2 (dynamically linked) libraries (DLLs) for Windows makes it possible to compile *Coot* without the requirement of the X11 windowing system, which would depend on an emulation layer (*e.g. Cygwin*). Some minor adjustments to *Coot* itself were necessary owing to differences in operating-system architecture, *e.g.* the filesystem (Lohkamp *et al.*, 2005 ▶). Currently *WinCoot*, by default, only uses Python as a scripting language since the Guile GTK+2 extension module is not seen as robust enough on Windows. *WinCoot* binaries are, as for GNU/Linux systems, automatically built and tested on a regular basis. The program is executed using a batch script and has been shown to work on Windows 98, NT, 2000, XP and Vista.

*WinCoot* binaries (stable as well as pre-releases) are available as a self-extracting file from http://www.ysbl.york.ac.uk/~lohkamp/coot/.

## 10. Discussion

*Coot* tries to combine modern methods in macromolecular model building and validation with concerns about a modern GUI application such as ease of use, productivity, aesthetics and forgiveness. This is an ongoing process and although improvements can still be made, we believe that *Coot* has an easy-to-learn intuitive GUI combined with a high level of crystallographic awareness, providing useful tools for the novice and experienced alike.

However, *Coot* has a number of limitations: NCS-averaged maps are poorly implemented, being meaningful only over a limited part of the unit cell (or crystal). There is also a mis­match in symmetry when using maps from cryo-EM data (*Coot* incorrectly applies crystal symmetry to EM maps). *Coot* is not at all easy to compile, having many dependencies: this is a problem for developers and advanced users.

### 10.1. Future

*Coot* is under constant development. New features and bug fixes are added on an almost daily basis. It is anticipated that further tools will be added for validation, nucleotide and carbohydrate model building, as well as for refinement. Interactive model building will be enhanced by communication with the *CCP*4 database, use of annotations and an interactive notebook and by adding annotation representation into the validation graphs. The embedded scripting languages provide the potential for sophisticated communication with model-building tools such as *Buccaneer*, *ARP*/*wARP* and *PHENIX*; in future this may be extended to include density modification as well.

In the longer term tools to handle EM maps are planned, including the possibility of building and refining models. The appropriate data structures are already implemented in the Clipper libraries but are not yet available in *Coot*.

The integration of validation tools will be expanded, especially with respect to *MolProbity*, and an interface to the *WHAT_CHECK* validation program (Hooft *et al.*, 1996 ▶) will be added. *WHAT_CHECK* provides machine-readable output and this can be read by *Coot* to provide both an interactive description and navigation as well as (requiring more work) a mode to automatically fix up problematic geometry.

*Note added in proof*: Ian Tickle has noted a potential problem with the calculation of χ^2^ values resulting from real-space refinement. *Coot* will be reworked to instead represent the r.m.s. deviation from ideality of each of the geometrical terms.

## Figures and Tables

**Table 1:** Map data labels output by common crystallography software, including *DM* (Zhang *et al.*, 1997 ▶), *Parrot* (Cowtan, 2010 ▶), *Pirate* (Cowtan, 2000 ▶) and *autoSHARP* (Vonrhein *et al.*, 2007 ▶)

Source of MTZ	Column labels for ‘best’ map
*DM*	FDM, PHIDM
*Parrot*	parrot.F_phi.F, parrot.F_phi.phi
*Pirate*	pirate.F_phi.F, pirate.F_phi.phi
*autoSHARP* (phasing)	FP, PHIB (or Fcent, PHIcent if light atoms only)
*autoSHARP* (post-*SOLOMON*)	FBsol, PHIBshasol (or Fcentshasol, PHIcentshasol if light atoms only)

## References

[bb1] Adams, P. D., Grosse-Kunstleve, R. W., Hung, L.-W., Ioerger, T. R., McCoy, A. J., Moriarty, N. W., Read, R. J., Sacchettini, J. C., Sauter, N. K. & Terwilliger, T. C. (2002). *Acta Cryst.* D**58**, 1948–1954. 10.1107/s0907444902016657 12393927

[bb2] Bernstein, F. C., Koetzle, T. F., Williams, G. J. B., Meyer, E. F. Jr, Brice, M. D., Rodgers, J. R., Kennard, O., Shimanouchi, T. & Tasumi, M. (1977). *J. Mol. Biol.* **112**, 535–542. 10.1016/s0022-2836(77)80200-3 875032

[bb3] Blanc, E., Roversi, P., Vonrhein, C., Flensburg, C., Lea, S. M. & Bricogne, G. (2004). *Acta Cryst.* D**60**, 2210–2221. 10.1107/S0907444904016427 15572774

[bb4] Blundell, T. L., Jhoti, H. & Abell, C. (2002). *Nature Rev. Drug Discov.* **1**, 45–54. 10.1038/nrd706 12119609

[bb5] Brünger, A. T. (1992). *X-PLOR* v.3.1. *A System for X-ray Crystallo­graphy and NMR.* New Haven: Yale University Press.

[bb6] Cowtan, K. (2000). *Acta Cryst.* D**56**, 1612–1621. 10.1107/s0907444900013263 11092927

[bb7] Cowtan, K. (2003). *IUCr Comput. Commun. Newsl.* **2**, 4–9.

[bb8] Cowtan, K. (2006). *Acta Cryst.* D**62**, 1002–1011. 10.1107/S0907444906022116 16929101

[bb9] Cowtan, K. (2010). *Acta Cryst.* D**66**, 470–478. 10.1107/S090744490903947X PMC2852311 20383000

[bb10] Dauter, Z. (2006). *Acta Cryst.* D**62**, 1–11. 10.1107/S0907444905034050 16369088

[bb11] Davis, I. W., Leaver-Fay, A., Chen, V. B., Block, J. N., Kapral, G. J., Wang, X., Murray, L. W., Arendall, W. B. III, Snoeyink, J., Richardson, J. S. & Richardson, D. C. (2007). *Nucleic Acids Res.* **35**, W375–W383. 10.1093/nar/gkm216 PMC1933162 17452350

[bb12] DeLano, W. L. (2002). *The PyMOL Molecular Viewer.* http://www.pymol.org.

[bb13] Emsley, P. & Cowtan, K. (2004). *Acta Cryst.* D**60**, 2126–2132. 10.1107/S0907444904019158 15572765

[bb14] Esnouf, R. M. (1997). *Acta Cryst.* D**53**, 665–672. 10.1107/S0907444997005829 15299854

[bb15] Greer, J. (1974). *J. Mol. Biol.* **82**, 279–301. 10.1016/0022-2836(74)90591-9 4817788

[bb16] Hooft, R. W. W., Vriend, G., Sander, C. & Abola, E. E. (1996). *Nature (London)*, **381**, 272. 10.1038/381272a0 8692262

[bb18] Jones, T. A. & Thirup, S. (1986). *EMBO J.* **5**, 819–822. 10.1002/j.1460-2075.1986.tb04287.x PMC1166864 3709525

[bb17] Jones, T. A., Zou, J.-Y., Cowan, S. W. & Kjeldgaard, M. (1991). *Acta Cryst.* A**47**, 110–119. 10.1107/s0108767390010224 2025413

[bb19] Kelsey, R., Clinger, W. & Rees, J. (1998). *Higher-Order and Symbolic Computation*, **11**, 7–105.

[bb20] Kleywegt, G. J. (1996). *Acta Cryst.* D**52**, 842–857. 10.1107/S0907444995016477 15299650

[bb21] Kleywegt, G. J., Harris, M. R., Zou, J., Taylor, T. C., Wählby, A. & Jones, T. A. (2004). *Acta Cryst.* D**60**, 2240–2249. 10.1107/S0907444904013253 15572777

[bb22] Kleywegt, G. J. & Jones, T. A. (1994). *Proceedings of the CCP4 Study Weekend. From First Map to Final Model*, edited by S. Bailey, R. Hubbard & D. Waller, pp. 59–66. Warrington: Daresbury Laboratory.

[bb23] Kleywegt, G. J. & Jones, T. A. (1996). *Structure*, **4**, 1395–1400. 10.1016/s0969-2126(96)00147-5 8994966

[bb24] Krissinel, E. & Henrick, K. (2004). *Acta Cryst.* D**60**, 2256–2268. 10.1107/S0907444904026460 15572779

[bb25] Krissinel, E. B., Winn, M. D., Ballard, C. C., Ashton, A. W., Patel, P., Potterton, E. A., McNicholas, S. J., Cowtan, K. D. & Emsley, P. (2004). *Acta Cryst.* D**60**, 2250–2255. 10.1107/S0907444904027167 15572778

[bb26] Langer, G., Cohen, S. X., Lamzin, V. S. & Perrakis, A. (2008). *Nature Protoc.* **3**, 1171–1179. 10.1038/nprot.2008.91 PMC2582149 18600222

[bb27] Lohkamp, B., Emsley, P. & Cowtan, K. (2005). *CCP4 Newsl.* **42**, contribution 7.

[bb28] Lorensen, W. E. & Cline, H. E. (1987). *ACM SIGGRAPH Comput. Graph.* **21**, 163–169.

[bb29] Lovell, S. C., Davis, I. W., Arendall, W. B. III., de Bakker, P. I. W., Word, J. M., Prisant, M. G., Richardson, J. S. & Richardson, D. C. (2003). *Proteins*, **50**, 437–450. 10.1002/prot.10286 12557186

[bb30] Lovell, S. C., Word, J. M., Richardson, J. S. & Richardson, D. C. (2000). *Proteins*, **40**, 389–408. 10861930

[bb31] Merritt, E. A. & Bacon, D. J. (1997). *Methods Enzymol.* **277**, 505–524. 10.1016/s0076-6879(97)77028-9 18488322

[bb32] Murshudov, G. N., Vagin, A. A. & Dodson, E. J. (1997). *Acta Cryst.* D**53**, 240–255. 10.1107/S0907444996012255 15299926

[bb33] Oldfield, T. J. (2001). *Acta Cryst.* D**57**, 696–705. 10.1107/s0907444901003894 11320310

[bb34] Persistence of Vision Pty Ltd (2004). *POV-Ray – Persistence of Vision Raytracer.* http://www.povray.org.

[bb35] Potterton, L., McNicholas, S., Krissinel, E., Gruber, J., Cowtan, K., Emsley, P., Murshudov, G. N., Cohen, S., Perrakis, A. & Noble, M. (2004). *Acta Cryst.* D**60**, 2288–2294. 10.1107/S0907444904023716 15572783

[bb36] Robertson, M. P. & Scott, W. G. (2007). *Science*, **315**, 1549–1553. 10.1126/science.1136231 17363667

[bb37] Robertson, M. P. & Scott, W. G. (2008). *Acta Cryst.* D**64**, 738–744. 10.1107/S0907444908011578 PMC2507861 18566509

[bb38] Storoni, L. C., McCoy, A. J. & Read, R. J. (2004). *Acta Cryst.* D**60**, 432–438. 10.1107/S0907444903028956 14993666

[bb39] Terwilliger, T. C., Klei, H., Adams, P. D., Moriarty, N. W. & Cohn, J. D. (2006). *Acta Cryst.* D**62**, 915–922. 10.1107/S0907444906017161 PMC2745883 16855309

[bb40] Vagin, A. A., Steiner, R. A., Lebedev, A. A., Potterton, L., McNicholas, S., Long, F. & Murshudov, G. N. (2004). *Acta Cryst.* D**60**, 2184–2195. 10.1107/S0907444904023510 15572771

[bb41] Vonrhein, C., Blanc, E., Roversi, P. & Bricogne, G. (2007). *Methods Mol. Biol.* **364**, 215–230. 10.1385/1-59745-266-1:215 17172768

[bb42] Wang, J. W., Chen, J. R., Gu, Y. X., Zheng, C. D., Jiang, F., Fan, H. F., Terwilliger, T. C. & Hao, Q. (2004). *Acta Cryst.* D**60**, 1244–1253. 10.1107/S0907444904010674 15213386

[bb43] Westbrook, J. D., Yang, H., Feng, Z. & Berman, H. M. (2005). *International Tables for Crystallography*, Vol. *G*, edited by S. R. Hall & B. McMahon, pp. 539–543. Heidelberg: Springer.

[bb44] Williams, S. P., Kuyper, L. F. & Pearce, K. H. (2005). *Curr. Opin. Chem. Biol.* **9**, 371–380. 10.1016/j.cbpa.2005.06.007 16006182

[bb45] Word, J. M., Lovell, S. C., LaBean, T. H., Taylor, H. C., Zalis, M. E., Presley, B. K., Richardson, J. S. & Richardson, D. C. (1999). *J. Mol. Biol.* **285**, 1711–1733. 10.1006/jmbi.1998.2400 9917407

[bb46] Word, J. M., Lovell, S. C., Richardson, J. S. & Richardson, D. C. (1999). *J. Mol. Biol.* **285**, 1735–1747. 10.1006/jmbi.1998.2401 9917408

[bb47] Zhang, K. Y. J., Cowtan, K. & Main, P. (1997). *Methods Enzymol.* **277**, 53–64. 10.1016/s0076-6879(97)77006-x 18488305

